# Cannabis, a cause for anxiety? A critical appraisal of the anxiogenic and anxiolytic properties

**DOI:** 10.1186/s12967-020-02518-2

**Published:** 2020-10-02

**Authors:** Lara Sharpe, Justin Sinclair, Andrew Kramer, Michael de Manincor, Jerome Sarris

**Affiliations:** 1 NICM Health Research Institute, Western Sydney University, Locked Bag 1797, Penrith, Westmead, NSW 2145 Australia; 2 Department of Psychiatry, The Melbourne Clinic, Professorial Unit, The University of Melbourne, Melbourne, Australia

**Keywords:** THC, CBD, Cannabinoid, Cannabis, Anxiety, Anxiolytic, Anxiogenic, Anti-anxiety

## Abstract

**Background:**

Cannabis has been documented for use in alleviating anxiety. However, certain research has also shown that it can produce feelings of anxiety, panic, paranoia and psychosis. In humans, Δ^9^-tetrahydrocannabinol (THC) has been associated with an anxiogenic response, while anxiolytic activity has been attributed mainly to cannabidiol (CBD). In animal studies, the effects of THC are highly dose-dependent, and biphasic effects of cannabinoids on anxiety-related responses have been extensively documented. A more precise assessment is required of both the anxiolytic and anxiogenic potentials of phytocannabinoids, with an aim towards the development of the ‘holy grail’ in cannabis research, a medicinally-active formulation which may assist in the treatment of anxiety or mood disorders without eliciting any anxiogenic effects.

**Objectives:**

To systematically review studies assessing cannabinoid interventions (e.g. THC or CBD or whole cannabis interventions) both in animals and humans, as well as recent epidemiological studies reporting on anxiolytic or anxiogenic effects from cannabis consumption.

**Method:**

The articles selected for this review were identified up to January 2020 through searches in the electronic databases OVID MEDLINE, Cochrane Central Register of Controlled Trials, PubMed, and PsycINFO.

**Results:**

Acute doses of CBD were found to reduce anxiety both in animals and humans, without having an anxiogenic effect at higher doses. Epidemiological studies tend to support an anxiolytic effect from the consumption of either  CBD or THC, as well as whole plant cannabis. Conversely, the available human clinical studies demonstrate a common anxiogenic response to THC (especially at higher doses).

**Conclusion:**

Based on current data, cannabinoid therapies (containing primarily CBD) may provide a more suitable treatment for people with pre-existing anxiety or as a potential adjunctive role in managing anxiety or stress-related disorders. However, further research is needed to explore other cannabinoids and phytochemical constituents present in cannabis (e.g. terpenes) as anxiolytic interventions. Future clinical trials involving patients with anxiety disorders are warranted due to the small number of available human studies.

## Background

*Cannabis spp.* have over 500 phytochemicals documented, including well over 100 cannabinoids, which are unique to the genus [1, 2]. Until recently, cannabis and its components were largely restricted under international legislation due to the perceived lack of medical value and the substantial risk of misuse [2]. As a result, the pharmacology of most of the cannabinoids are largely unknown. However, one of the more potent psychoactive compounds, Δ^9^-tetrahydrocannabinol (THC), has been extensively isolated, synthesised and studied [3] since it was first isolated in 1964 [4]. Along with the emergence of literature on this compound, there has been a corresponding increase in the use of cannabis for medical purposes, with the most frequently stated reasons for its use being for the management of pain, anxiety and depression [5].

Cannabis remains the most commonly consumed illicit drug around the world [6], whilst clinical research is nascent, yet rapidly emerging. Research is urgently required due to the large variety of cannabis preparations that are available on both the licit and illicit drug markets (depending on jurisdictions) [1]. Furthermore, both community and laboratory-based studies have demonstrated that the relative quantities of cannabinoids in the plant may directly affect its pharmacological activity when consumed. For example, when taken together with THC, CBD may potentially offset some of the adverse effects of THC, such as memory impairment and paranoia [7, 8]. It has been demonstrated in rodents that high doses of CBD are able to negate some of the anxiogenic response created by THC [9].

Recreational use of cannabis is commonly reported to lead to a feeling of euphoria accompanied by a decrease in anxiety and an increase in sociability [10]. Conversely, it is also frequently reported that cannabis can produce feelings of anxiety, panic, paranoia and psychosis [3, 11–16]. It has also been demonstrated that changes in sociability depends on prior exposure and use of cannabis [17]. So why may this contradictory finding be present? Studies have indicated that the two predominant compounds in cannabis: CBD and THC, appear to have opposing actions, with the reported anxiolytic effect attributed to CBD and anxiogenic outcomes being attributed to the THC [18]. Nevertheless, a number of more recent publications have shown that this outcome of THC is dosage-dependent, with lower dosages having the opposite effect.

**Fig. 1:**
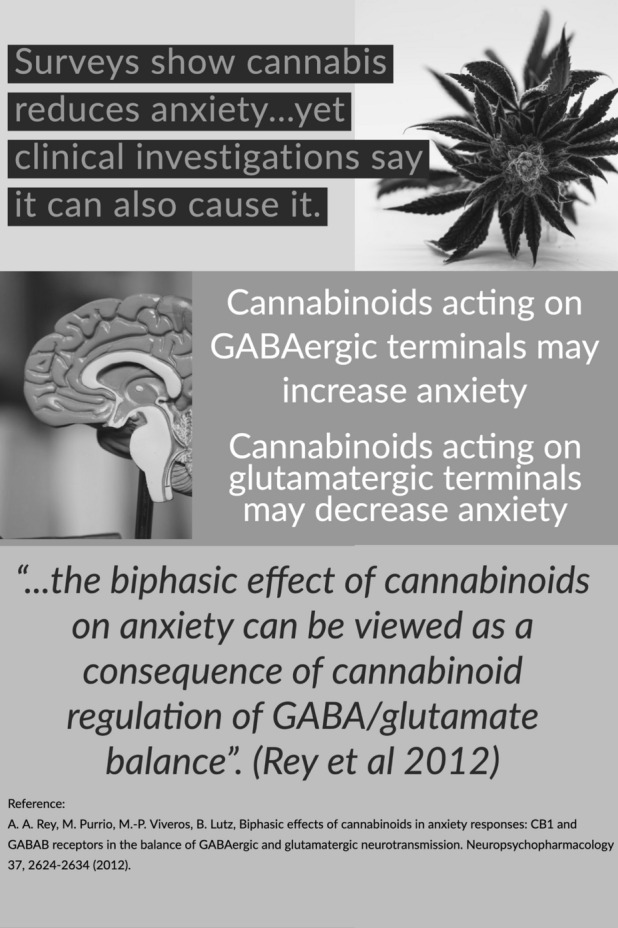
Summary of biphasic anxiolytic/anxiogenic effects of cannabis

There is extensive research supporting the biphasic nature of cannabinoids in both anxiety [19–25] (Fig. 1) and behavioral responses including motor activity [26–30] and aggression [31]. Different doses of THC have been found to be biphasic in reward and motor activity [32], and memory and cognition [31, 33]. Whilst the majority of these studies have been conducted on rodents, human studies (covered in detail later) have also provided promising results. Several reports have also found that in animals [34–37], as well as in humans [37], THC acts differently according to whether it is administered by itself or concurrently with other cannabinoids or terpenes. It has been discussed in the literature that CBD, due to its anxiolytic properties, may have a protective effect against certain negative psychological effects from THC [7, 8]. Research has also shown that it may also be capable of antagonising at least some of the adverse effects related to THC [1, 38]. Recent research has indicated that when low-dose CBD (4 mg) is combined with THC the intoxicating effects of THC were enhanced, while high doses of CBD (400 mg) decreased the same effects [38]. Furthermore, the plethora of chemical constituents found in whole cannabis have been found to be more active than single, purified phytocannabinoids [4, 39]. This being said, cannabis terpenoids as potential synergistic contributors to the effects of phytocannabinoids has not yet been explored in sufficient detail [39].

The plant’s anxiety-modulating action has largely been attributed to a biphasic interaction with the CB1 receptor. Rey et al. (2012) [40] found that the anxiolytic effects of low doses occur when they interact with the CB1 receptor on cortical glutamatergic terminals. Conversely, interaction with the CB1 receptor on the GABAergic terminals is responsible for anxiogenesis, something which takes place when higher doses are administered. Further, the use of a CB1 receptor antagonist has been found to fully reverse the effects of THC [41]. However, other non-CB1 receptors are also believed to be involved including serotonin 5-HT1A receptors [42] and the opioid system [20, 43, 44]. There has also been research in recent years to determine the neural site at which these interactions take place. These studies have largely involved injecting THC into various parts of the brain in animal models and observing any anxiolytic or anxiogenic effect [41]; or by observing the effects of oral doses on the brains of individuals under the influence of THC using functional magnetic resonance imaging (fMRI) [45]. Not surprisingly, it has also been found that an individual’s history of cannabis use plays a role in the response of an individual to cannabis intake [46], something which has been observed in both animal [20, 42, 43, 47] and human models [48].

Whilst other papers have reviewed the association of cannabis with anxiety prevalence [49], or explored the underlying potential anxiolytic or anxiogenic mechanisms of action [20, 41–45], or covered the current human clinical trial evidence in the area [16], no comprehensive integrated paper exists to date which critically appraises both the potential anxiolytic and anxiogenic effects of the plant across these research domains. This review seeks to fill this void by compiling a broad overview of the scientific literature on both the anxiolytic and angiogenic properties of both whole plant cannabis and isolates (e.g. THC, CBD, and other phytocannabinoids and terpenes) in both animals and humans. This systematic review covers animal models, epidemiological data and human clinical trials, concluding with a perspective for industry, clinicians, and the public about current recommendations for medicinal cannabis formulations which may provide anxiolytic activity with lesser risk of anxiogenic effects.

## Method

To provide a comprehensive review of the area, both animal and human studies were sought for inclusion. In order to include as many relevant sources as possible, there were no exclusions based on types of animals or models (testing anxiety or mood paradigms) used in the studies. Human studies included in the review involved either epidemiological studies exploring the cross-sectional or longitudinal association between cannabis use and anxiety, or interventional studies using whole cannabis extracts or isolates (botanically-derived only) for any anxiety disorder, or to test an acute anxiogenic or anxiolytic effect. Synthetic cannabinoid analogues were excluded from this review.

**Fig. 2:**
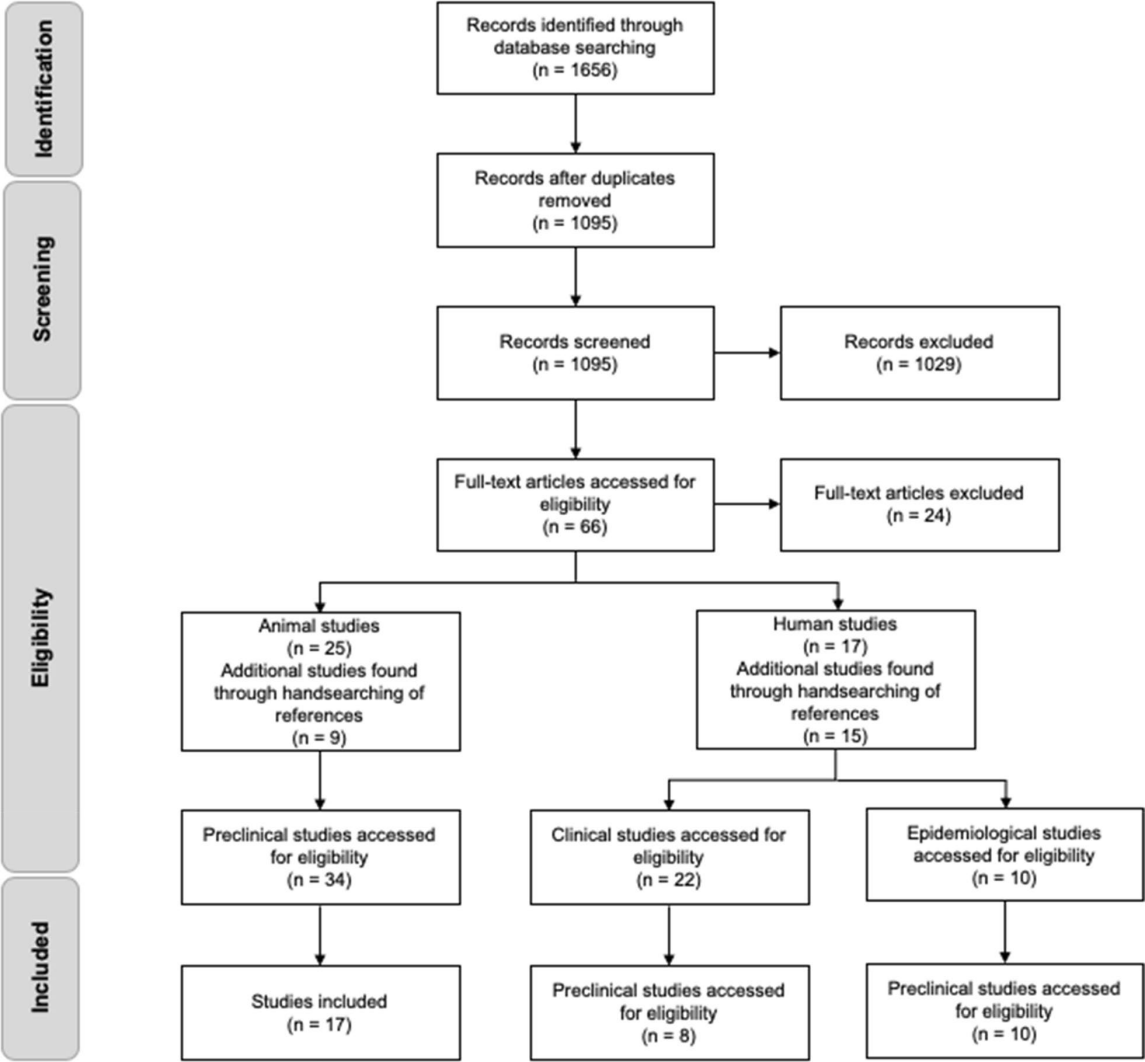
Process of identification and screening of articles for inclusion

Articles were identified using the electronic databases of OVID MEDLINE, Cochrane Central Register of Controlled Trials, PubMed, and PsycINFO up to January 2020, and only included articles in English. No time limits were set. Intervention studies (animal or human) could involve either acute or chronic administration of cannabis-based treatment. Studies testing major cannabinoids or whole plant interventions were included. Where the composition was unknown, studies where THC was administered via cigarette or inhaler were excluded for the clinical trial portion of this review. In addition, reference lists were searched for additional references. The main database search was split into three systematic search streams: animal models; epidemiological data; human clinical trials (see Fig. 2). An additional limit was set for epidemiological studies over the past five years (2016-2020), due to the breadth of current data. The term ‘significant’ was used for a *p* value of < 0.05.

The following search terms were used to locate animal models as well as epidemiological and intervention studies:

“delta 9 thc OR THC OR tetrahydrocannabinol OR delta 9 tetrahydrocannabinol OR delta 9-THC OR D9-THC OR Delta [9] -THC OR Δ9-THC OR CBD OR canna* OR terpenes AND “anxi* OR anxiety disorder* OR anxiolytic* OR anti-anxiety OR anxiogenic OR social phobia OR social anxiety OR panic disorder OR post-traumatic stress disorder OR PTSD.

Our search revealed a total of 1095 studies with 66 being relevant for a full review of the articles for potential inclusion. A final review revealed a total of 35 studies eligible for inclusion (17 preclinical, 8 human, and 10 epidemiological).

## Results

### Epidemiological data

Our review of the data revealed 10 studies involving cannabis users consuming whole cannabis preparations or extracts for anxiety (see Tables 1, 2). Included in our review were cross-sectional studies with no demographic limitations. Three studies in particular demonstrated that such use is prevalent, with more than half of the participants in each survey confirming using cannabis for anxiety [50–52]. Further, these studies indicate that there is also a significant proportion of people who replaced some or all prescription medication with cannabis use [53, 54]. The majority of participants were recruited online, particularly through social media or through medicinal cannabis suppliers.

**Table 1:** Epidemiological studies of whole cannabis for anxiety (part 1)

Study	Data source	Number of participants	Route of administration	Dosage	Percent of respondents using for anxiety
Sexton et al. 2016 [50]	Recruited via social media and Cannabis dispensaries in Washington State	1429	Inhalation (84.1%), Concentrates also typically inhaled (6.4%), Oral (8%), Topical: (0.6%), Fresh juice (0.5%), Other (0.4%)	47.6% reported using 1–4 times per day 14.9% reported using 5–10 times per day 12.2% reported using all day, every day. 25.3% reported using less than once a day Dose is N/A	58.1%
Lintzeris et al. 2018 [51]	Recruited via online media and at professional and consumer forums	1748	Most frequent route was inhalation (83.4%) Oral Topical, suppository, and vaporiser routes used by only 29.4% of respondents	N/A	51%
Turna et al. 2019 [56]	An online survey was disseminated to all authorized CMP users registered with Canadian medicinal cannabis supplier Tilray	2032	47.6% reported vaporising as the preferred mode of delivery, 21.4% preferred oral ingestion (edibles, oils, etc.) 18.5% preferred joints	35% used < 1 g/day 42% used 1–2 g/day 23% used ≥ 3 g/day.	43.7%
Kosiba et al. 2019 [52]	Data were extracted from 13 studies that included participants from over 30 countries	6665	N/A	N/A	52%

**Table 2:** Epidemiological studies of whole cannabis and cannabis extracts for anxiety (part 2)

Study	Data Source	Number	Route of Administration	Outcome
Cuttler et al. 2016 [59]	Recruited via word-of-mouth and links on advertisements posted on various websites and in Washington State cannabis dispensaries	1418 participants	Inhaled- (joints, bong, pipe, vaporiser) – (M) 89.8%, (F) 88% Oral- (M) 3.9%, (F) 7.9% Concentrates- (M) 5.4%, (F) 3.1% Topical- (M & F) 0.4% Other- (M) 0.6%, (F) 0.73%	Male: 55.3% Female: 57.2% Reported feeling less anxious or fearful
Corroon et al. 2017 [53]	Recruited via social media	2774 participants	N/A	46% reported using cannabis as a substitute for prescription drugs
Piper et al. 2017 [54]	New England dispensary members	1513 participants	N/A	71.8% reduced medication prescribed for anxiety
Corroon et al. 2018 [55]	Recruited via social media.	2409 participants	N/A	Almost 62% of CBD users reported using CBD to treat a medical condition. The top three medical conditions were pain, anxiety, and depression
Feingold et al. 2018 [58]	Data was drawn from Waves 1 and 2 of the National Epidemiologic survey on Alcohol and Related Conditions	3723 participants	N/A	Remission rates for non-users: 66.0% Remission rates for users: 52.8%)
Cuttler et al. 2018 [57]	Data from the cannabis tracking app Strainprint^TM^	5085 tracking sessions	Inhalation (smoking, vaping, concentrates, dab bubbler, dab portable)	93.5% of sessions recorded decrease in anxiety

The three cross-sectional studies found that respondents reported that they used cannabis medicinally for anxiety, second only to pain [50, 52, 55], with close to half of all survey participants stating they use cannabis for anxiety [50–52, 56]. In a study of 1429 participants, Sexton et al. (2016) [50] found that over half (59.8%) of medical users reported using cannabis as an alternative to pharmaceutical prescriptions [50]. Similarly, a US study of 2774 participants found this to be 46% of users [53]. Additionally, one study of 2032 people found that nearly half of the respondents had substituted an anxiety medication prescribed to them by their physician, with medical cannabis [56], and 61% indicated that cannabis had completely replaced their prescribed medication. Likewise, another study consisting of 1513 participants found similar results, with 71.8% indicating that they had reduced their intake of anti-anxiety medications [54] (Table 2).

In a review of 5085 responses recorded in a smart-phone application, it was found that users of the app reported significantly lower anxiety levels following cannabis use [57] (Table 2). Further, only 2.1% experienced exacerbated symptoms, while only 4.4% reported no change in anxiety symptoms. An Australian study of 1748 participants found that fewer than 1% of respondents felt that the treated symptom, including anxiety, had worsened compared to 71 to 92% who felt it had improved [51]. Such results were further confirmed by Turna et al. (2019) [56] where 92% of the 2032 respondents reported that cannabis improved their anxiety symptoms. Despite this response, the scores of self-reported questionnaires indicate that symptoms remained moderately severe.

In a 3-year longitudinal survey of cannabis use by patients with a primary anxiety disorder diagnosis (N = 3723), it was found that remission rates from anxiety disorders were higher among cannabis nonusers (Table 2). However, these differences were not statistically significant in adjusted models [58]. Discrepancies in responses are further highlighted as men reported experiencing greater headache/migraine relief from medical cannabis than women, despite a larger proportion of women reporting using it for this reason. Of note also is that women were significantly more likely than men to report using cannabis to treat anxiety [59]. A summary caveat concerns that the epidemiological data should be considered within the limitation of survey respondents being a ‘captive’ sample who had an active interest in cannabis use.

### Animal studies

Our initial search returned 1095 articles, with a further nine studies found through handsearching of the references. A total of 17 preclinical studies were found to be relevant for inclusion (Tables 3 and 4). The focus of the research concerned primarily CBD and/or THC.

**Table 3:** Reviews of CBD’s anxiolytic properties in rats and mice

Study	Conclusion
de Mello Schier et al. 2012 [60]	This reviewed included 17 studies. One study reviewed showed no significant effects of high doses of CBD (100 mg/kg) were seen in rats in the Geller-Seifter conflict test. In another, a low dose of CBD (10 mg/kg) had anxiolytic effects in rats submitted to the conditioned emotional response. Later studies using the elevated plus maze (EPM) helped to elucidate this contradiction. The authors concluded that anxiolytic effects are only present at low doses
Blessing et al. 2015 [61]	This review included 32 studies. The authors concluded that overall, preclinical evidence supports systemic CBD as an acute treatment of GAD, SAD, PD, OCD, and PTSD, and suggests that CBD has the advantage of not producing anxiogenic effects at higher dose
Lee et al. 2017 [62]	This review included 28 studies of both acute and chronic CBD administration on anxiety-like behaviour in male animals. The majority of the listed studies were in Wistar rats, and predominantly, anxiolytic effects were noted
Iffland and Grotenhermen 2017 [63]	The authors observed in the data that anxiolytic effects in rats were reversed after repeated 14-day administration of CBD. However, this finding might depend on the used animal model of anxiety or depression and that CBD may only be anxiolytic in subjects where stress had been induced before CBD administration

**Table 4:** Studies of the anxiogenic/anxiolytic effect of THC and other cannabinoids in animals

Study	Animal	Compound	Model	Route	Anxiolytic/Anxiogenic-like effect?
Acute administration					
McLendon et al.1976 [65]	Male Rhesus monkeys	THC (0.2, 0.5, 1.0 mg/kg)	Cardiac conditioned response	Intravenous	All does produced an anxiolytic-like effect
Valjient et al. 2002 [21]	Male CD-1 mice	THC (0.03, 0.1, 1.0, 2.5, 5.0 mg/kg)	Light–dark (LD) box	Intraperitoneal	The lowest dose of 0.03 mg/kg produced an anxiolytic-like effect and the highest dose of (5.0 mg/kg) produced an anxiogenic-like effect. All other doses produced no effect.
Berrendero and Maldonado 2002 [20]	Male CD1 mice	THC (0.3 mg/kg)	Light–dark box	Intraperitoneal	The dose of 0.3 mg/kg produced an anxiolytic-like response
Schramm-Sapyta et al. 2007 [68]	Adolescent and adult Male CD rats	THC (0.5, 2.5 mg/kg)	Light–dark box, Elevated plus-maze (EPM)	Intraperitoneal	All doses produced anxiogenic-like responses
Braida et al. 2007 [42]	Male Sprague–Dawley rats	THC (0.015, 0.075, 0.75 mg/kg)	Elevated plus-maze	Intraperitoneal	All doses produced anxiolytic-like responses
Rubino et al. 2007 [67]	Male Sprague–Dawley rats	THC (0.015, 0.075, 0.374, 0.75, 1.5, 3.0 mg/kg)	Elevated plus-maze	Intraperitoneal	At all doses except 3.0 mg/kg, an anxiolytic-like response was observed. When 3.0 mg/kg was administered, no response was seen.
Long et al. 2010 [69]	Male C57BL/6JArc mice	CBD (1.0, 50.0 mg/kg)	Elevated plus-maze	Intraperitoneal	No effect was observed at either dose
Fokos et al. 2010 [64]	Male Sprague–Dawley rats	THC (0.5, 1.0 mg/kg)	Elevated plus-maze	Intraperitoneal	In stressed rats, 0.5 mg/kg THC produced an anxiogenic-like effect and 1 mg/kg produced an anxiolytic-like effect. On the other hand, in non-stressed rats, both doses produced an anxiolytic-like response
Kasten et al. 2019 [72]	Adolescent and adult male and female C57Bl/6 J mice	THC (1.0, 5.0, 10.0 mmg/k) CBD (5.0, 10.0, 20.0 mg/kg) THC + CBD (10.0 + 20.0 mg/kg)	Elevated plus-maze, Open field (OF) test	Intraperitoneal Male and Female, Adult and Adolescent mice (we have looked ta adult mice only in this section)	In the elevated plus maze and the open field test, all doses of THC produced an anxiogenic-like response. For both tests, all doses of CBD produced no effect. When the combination of THC and CBD was given, an anxiogenic-like response was observed in both the elevated plus maze and the open field test
Zieba et al. 2019 [71]	Male Fmr1 KO mice	CBD (5.0, 20.0 mg/kg)	Elevated plus-maze, Open field test	Intraperitoneal	In the EPM, both 5.0 and 20.0 mg/kg CBD produced an anxiolytic-like response. On the other hand, in the OF test, no effect was observed at either dose
Malone et al. 2009	Male Sprague–Dawley rats	CBD (5.0, 20.0 mg/kg) THC (1.0, 3.0, 10.0 mg/kg) THC + CBD (1.0 + 5.0 mg/kg, 1.0 + 20.0 mg/kg, 3.0 + 5.0 mg/kg, 3.0 + 20.0 mg/kg, 10.0 + 5.0 mg/kg, 10.0 + 20.0 mg/kg	Social Interaction	Intraperitoneal	All dose effects are comparative to each other (see results discussion above)
Chronic administration					
Long et al. 2010 [69]	Male C57BL/6JArc mice	THC (0.3, 1.0, 3.0, 10.0 mg/kg) CBD 1.0, 5.0, 10.0, 50.0 mg/kg)	Light–dark box, Elevated plus-maze, Open-field test, Social interaction	Intraperitoneal	At 10 mg/kg THC, there was an increase in the time spent in the light and the distance ratio. Further, There was a trend towards an effect of on time spent in the inner open arm in the EPM but no effect on the open-arm in the EPM. In the OF test, there was a significantly decrease in the time spent in the central zone of the on test on day 15 and decreased the distance ratio on day one. THC did not alter total SI time, but decreased the combined frequency of the social behaviors. In the LD test, CBD (1 mg/kg) significantly increased the time spent in the light compartment and the distance ratio. No effect on the open-arm entry ratio or percentage of time spent on open arms in the EPM. There was an Increase in the time spent in the central zone of the OF test on day 15.
Rock et al. 2017 [73]	Male Sprague–Dawley rats	THC (1.0, 10.0 mg/kg) CBD (5.0 mg/kg)	Light–dark box	Intraperitoneal	At a dose of 1 or 10 mg/kg, THC decreased the amount of time spent in the light box on days one and 21, suggesting an anxiogenic-like effect. As well, at a dose of10 mg/kg only, THC increased the latency to enter the light box, but only on day one. The addition of prior stress increased thus anxiogenic effect A prior stressor produced anxiogenic-like behavior, but administration of CBD abolished this effect
Schleicher et al. 2019 [70]	Male and Female C57BL/6 J mice	CBD (20.0 mg/kg)	Light–dark box, Open Field Test, Elevated Plus Maze	Intraperitoneal	No changes in anxiolytic/anxiogenic behavior was observed in the light–dark box and the open field test, but a decreased amount of time was spent in the open arms of the EPM
Direct administration into brain					
Rubino et al. 2008 [41]	﻿Male Sprague–Dawley rats	THC (0.0025, 0.005, 0.01, 0.025 mg into the prefrontal cortex, 0.0025, 0.005, 0.01 mg into the ventral hippocampus, 0.001, 0.0025, 0.005, 0.01 mg into the the basolateral amygdala	Elevated plus-maze	Microinjection	THC at doses of 0.0025 and 0.005 mg microinjected into the prefrontal cortex did not significantly affect anxiety behaviour, while 10 mg THC increase the percentage of time and entries onto the open arm, consistent with an anxiolytic effect. The anxiolytic-like response of 0.01 mg THC was also confirmed by the analysis of ethological parameters. At 0.025 mg the anxiolytic effect was lost in favour of an anxiogenic profile In the ventral hippocampus the dose of 0.005 mg THC significantly increased the percentage of open arm time as well as the percentage of open arm entries. This anxiolytic effect was confirmed by the significant decrease in the ethological parameter closed arm returns. In contrast, 0.01 mg THC seemed to switch to an anxiogenic response as shown by the reduction in open arm time and head dips, although not reaching statistical significance In the basolateral amygdala the dose of 0.001 mg induced a significant decrease in the percentage of open arm time and head dips and a tendency to decrease in the open arm entries, consistent with an anxiogenic-like response. Higher THC doses did not affect rat anxiety behaviour in the EPM. THC at all doses did not change closed arm entries

With respect to CBD, both Schier et al. (2012) [60] and Blessing et al. (2015) [61] concluded that when it was administered acutely, anxiolytic-like effects were only present at low doses, yet has the advantage of not producing anxiogenic effects at higher dose (see Table 3). Schier et al. (2012) [60] also noted that chronic doses produced mixed results, with both anxiolytic-like and anxiogenic-like outcomes being observed. Lee et al. (2017) [62] observed predominantly anxiolytic-like responses in the studies analysed, which applied to both acute and chronic administration. Iffland & Grotenhermen (2017) [63] concluded that CBD may only be anxiolytic where stress had been induced before CBD administration.

There was also some variance in the results. For example, Valjient et al. (2002) [21] observed that only the highest dose of 5.0 mg/kg had an anxiogenic-like effect, and lowest dose of 0.03 mg/kg had an anxiolytic-like effect in male CD-1 mice. Conversely, Fokos et al. (2010) [64] observed the opposite in male Sprague–Dawley rats with the low dose of 0.5 mg/kg producing an anxiogenic-like effect and the high dose of 1 mg/kg producing an anxiolytic-like effect. In McLendon et al.’s (1976) [65] study of male Rhesus monkeys, all doses from 0.2 mg/kg to 1 mg/kg produced an anxiolytic-like response. Conversely, Rock et al. (2017) [66] observed an anxiogenic-like response for both dosages of 1.0 mg/kg and 10 mg/kg in male Sprague–Dawley rats.

This variance may partly be due to different animals being studied. While McLendon et al. (1976) [65] used monkeys in their study, this was the only study found to do so, with the rest of the reviewed studies using rodents. Studies also differed in design, including types of test employed, the size of the apparatus used, dosages administered, and the route of administration.

### Elevated plus-maze (EPM)

Braida et al. (2007) [42] injected male Sprague–Dawley rats with a THC dosage of either 0.015, 0.075 or 0.75 mg/kg and then placed them in the EPM. It was found that THC exhibited a dosage-dependent effect with the highest dosage of THC corresponding to the maximum anxiolytic effect. Another approach involved male Sprague–Dawley rats being administered dosages ranging from 0.075 to 1.5 mg/kg [67]. It was found that even with the addition of a higher dosage compared to the previous study, the maximum anxiolytic effect was still found to occur when the rats were administered 0.75 mg/kg THC, which supports the idea that depending on the dose THC can produce both anxiolytic and anxiogenic responses. ﻿The study by Schramm-Sapyta et al. (2007) [68] was unique in that rats were used in their EPM instead of mice. These male CD rats were also divided into two age groups: adolescent and adult. The rats were injected with either 0.5 or 2.5 mg/kg THC. They concluded that while there was a significant effect of drug dose on the percentage of time spent in the open arms, there was no significant effect of age on this outcome. At the lower dose of 0.5 mg/kg though, THC was less anxiogenic in adolescents than in adult rats.

The next study sought to determine the brain regions involved in producing anxiogenic or anxiolytic effects by injecting THC ranging from 0.001 mg to 0.01 mg directly into various parts of the rat brain [41]. The results indicated that in certain regions, different dosages produce opposite effects. For example, when injected into the ventral hippocampus, the lower dose of 0.005 mg produced a significant anxiolytic-like effect, which switches to an anxiogenic-like response when 0.01 mg was injected. In contrast, low doses had no effect when injected into the prefrontal cortex, whereas the higher dose of 0.01 mg produced an anxiolytic like response and 0.025 mg produced an anxiogenic-like outcome. When injected into the basolateral amygdala, 0.001 mg THC induced a significant anxiogenic-like response whereas higher THC doses did not affect anxiety behavior.

In an alternative to the typical rat-model studies above, one study utilised male C57BL/6 JArc mice [69]. When CBD was administered acutely, there was no change in the percentage of time in the open arms or ratio of open-arm entries was observed. Neither was any change in the total number of EPM arm entries. In contrast, Schleicher et al. (2019) [70] found that in male and female ﻿C57BL/6J mice who were injected with 20 mg/kg CBD for 6 weeks there was a significant decrease in the time spent in the open arms [70]. Conversely Zieba et al. (2019) [71] found that acute administration of CBD increased time in open arms of EPM in male Fmr1 KO mice. The same mice were all given both doses with at least three days between tests. When given the higher dose (20 mg/kg), they were found to spend a longer amount of time in open arms compared to when they received the lower dose (5 mg/kg) (p < 0.005 and p < 0.05, respectively) [71].

In Long et al.’s (2010) [69] study of chronic administration, male C57BL/6JArc mice received 21 consecutive daily intraperitoneal injections of either THC (0.3, 1.0, 3.0 or 10.0 mg/kg) or CBD (1.0, 5.0, 10.0, 50.0 mg/kg). While there was a trend (p = 0.08) towards an effect of THC on time spent in the inner open arm, there was no effect on the open arm entry ratio. When CBD was administered, there was no effect on the open-arm entry ratio or percentage of time spent on open arms [69]. However, there was a similar trend (*p *= 0.09). towards an effect of CBD on time spent in the open arm section closest to the center zone of the EPM.

Like Schleicher et al. (2019) [70], Kasten et al. (2019) [72] also used C57Bl/6 J mice, but also included both sexes, and both adults and adolescents in their observations. The mice were injected with THC (1.0, 5.0 or 10.0 mg/kg), CBD (5.0, 10.0 or 20.0 mg/kg), and THC + CBD (10 mg/kg and 20 mg/kg respectively). Although there were no trends consistent across all categories, they did observe that while there was no significant effect of age there was a significant dose-related reduction in the time spent in open arms and open arm entries. Conversely it was observed that there was no interaction between the dose of CBD and the time spent on the open arms.

Another method saw male Sprague–Dawley exposed to either 10 days of chronic unpredictable stress or no stressor [64]. After this period, they were injected with either a low (0.5 mg/kg) or a high (1.0 mg/kg) dosage of THC, then being placed in an EPM. It was observed that in unstressed animals, the rats that were administered either 0.5 mg/kg or 1 mg/kg THC showed anxiolytic-like effects. In stressed animals, however, only the high dosage of THC induced an anxiolytic-like response, whereas the low dosage induced anxiogenic effects. These results directly contradict both the idea that THC is anxiolytic at low dosages, and anxiogenic at high dosages at least when stress is applied.

### Light-dark (LD) box

Although the aim of the Valjent et al. (2002) [21] study was to determine the effect of THC and nicotine administered together, we were able to utilise their results in this review, as THC was first administered alone. This involved the acute administration of either 0.03, 0.1, 0.3, 1, 2.5 or 5 mg/kg to determine at what dosage THC would produce a clear anxiolytic-like response. It was found that anxiolysis occurred at a dosage of 0.3 mg/kg. This markedly changed to an anxiogenic effect when 5.0 mg/kg was administered and there was no change in the response relative to vehicle for all other dosages given. These findings were further confirmed when in the same year the low dosage of 0.3 mg/kg THC was again given to male CD1 mice and once again an anxiolytic-like response was observed [20]. This was done based on the conclusions of the previous study, and with the intention to induce this anxiolytic-like response. Alternative dosages of 0.3, 1, 3 or 10 mg/kg were also employed [69]. The timeframe also differed, with these given in 21 daily injections. This study implies that there is a clear correlation between increasing dosages of THC and time spent in the dark area of the LD box.

In contrast to the other studies, Schramm-Sapyta et al. (2007) [68] looked at acute THC administration in adolescent and adult male CD rats. The rats received either 0.5 or 2.5 mg/kg THC. It was observed that the time in the light compartment was significantly reduced proportionally to increasing dose by THC in both adolescents and adults. Conversely, Rock et al. (2017) [73] studied the effect of THC chronic administration on male Sprague–Dawley rats using dosages of 1.0 and 10 mg/kg. At the dosages chosen, THC decreased the amount of time spent in the light chamber of the LD box on days one and 21, suggesting an anxiogenic-like effect both acutely as well as chronically. Furthermore, at a dose of 10 mg/kg only, THC increased the latency to enter the light box, but only on Day 1. This latency to enter was increased with the addition of a prior stressor. Long et al. (2010) [69] found that THC given at a high dose of 10 mg/kg to male C57BL/6JArc mice significantly decreased the time spent in the light compartment. Contrarily, it was observed that when the low dose of 1 mg/kg CBD was administered this resulted in a significant increase in the time spent in the light compartment. However, when 20 mg/kg CBD was given over a period of 6 weeks, no change in anxiety related behaviour was observed [70].

### Open field (OF) test

Long et al. (2010) [69] tested mice injected with THC in an OF test. The ratio of central to total distance travelled (distance ratio) and the time spent in the central zone were taken as measures of anxiety. It was noted that when the maximum dosage of 10 mg/kg was given, there was a significant decrease in the time spent in the central area and a decrease in the distance ratio. This was consistently demonstrated when THC was given daily over 21 days, with a significantly decreased overall distance travelled on day 15 and on day 21, the latter of which was also observed when doses of 1 mg/kg and 3 mg/kg were given.

Kasten et al. (2019) [72] found that 5 and 10 mg/kg doses of THC in adult mice reduced total locomotion. In the 5 mg/kg adult group this was significantly correlated with reduced time in the centre of the open field indicating an anxiogenic-like response. When 10 mg/kg CBD was given, reduced activity in the adult group was also observed, but this was not significantly correlated with anxiety-like metrics. In support of this Long et al. (2010) [69] observed that acute doses of CBD (1 and 50 mg/kg) produced an anxiolytic-like effect and Schleicher et al. (2019) [70], who injected male and female C57BL/6 J mice over a period of time, found that anxiety behaviour in the open field test was not affected. In contrast, Zieba et al. (2019) [71] found that in their male Fmr1 KO mice acute CBD treatment had no impact on anxiety related parameters in the open field test [71]. However, they did find that CBD given chronically at 50 mg/kg increased the time spent in the central zone of the OF test on day 15.

### Social interaction

The social interaction test for rodents was first introduced by File and Hyde (1978) [74]. In this study experimental manipulation was used to increase anxiety and this was observed to result in a decrease in social interaction. This test has continued to be used as it is sensitive to both anxiolytic and anxiogenic effects [75] and is an accepted measure of anxiety-like behaviours.

Test male C57BL/6JArc mice and those who had received 0.3, 1, 3 or 10 mg/kg THC were placed in opposite corners of a grey perspex arena to test social interaction [69]. Mice were allowed to explore freely for 10 min during which time the authors recorded manually the frequency and total duration of the active socio-positive behaviours undertaken by the mouse who had received the dosage of THC. It was found that while THC decreased the combined frequency of the socio-positive behaviours, the total duration of all these behaviours remained the same. However, the duration ﻿was decreased at 10 mg/kg THC, indicating an anxiogenic-like response at this higher dose.

Malone et al. (2009) [9], pre-treated male Sprague–Dawley rats with either vehicle, 5.0 or 20 mg/kg CBD. These rats were then administered either vehicle, 1.0, 3.0 or 10 mg/kg THC. A significant CBD-THC interaction was observed, as well as a significant effect of CBD on the total time spent interacting. The overall trend was that rats treated with a combination of a low dose of CBD and THC interacted less than rats treated with just the THC. However, when the dose of CBD was increased, these rats interacted more than those treated with just the THC. This outcome suggests that while CBD is able to negate some of the anxiogenic response of THC, higher doses of CBD are needed to achieve this.

#### Cardiac conditioned response (CCR)

McLendon et al. (1976) [65] used pairing one of two tones with the delivery of a peripheral electric shock in male Rhesus monkeys to establish the cardiac conditioned response (CCR). The conditioned response is considered to be part of the complex of physiological and behavioural changes characteristic of anxiety and has been used to study anxiety in human [65, 76]. The effect of various dosages of 0.2, 0.5 or 1.0 mg/kg intravenous THC was given. The results revealed that THC blocked the CCR in a dosage dependent manner and this was consistent across trials and across animals. At the lowest dosage tested of 0.2 mg/kg a slight attenuation was consistently noticed with a reduction in the conditioned response of 5 to 6 beats per minute observed. At the next highest dosage of 0.5 mg/kg a reduction of 10 to 15 beats per minute was noted for each animal and at the highest dosage of 1 mg/kg, there was a resultant complete block of the CCR in every case.

As detailed in Table 4, our search revealed a range of studies of cannabinoids (primarily THC) in anxiety models beginning in 1976. Research over this period of 40 + years has revealed conclusions that are inconsistent. Generally, the results indicate that at lower dosages an anxiolytic response for THC is observed, with the opposite being true of higher doses (however as indicate above across differing animal modes, this finding is not always consistent).

### Human studies

Of the initial 1095 articles detected in our initial search, 26 full text articles were assessed for eligibility. Of these, 17 met our initial inclusion criteria and an additional five were identified through handsearching of references. Of these, eight were found to meet inclusion criteria and are included in this review.

### Acute human clinical trials

The anxiogenic properties of isolated THC has have been firmly established in humans and as demonstrated in Table 5, and no human studies provided any evidence of anxiolytic effects. However, the dosages administered varied widely in the studies described ranging from 2.5 mg [48, 77] to 30 mg [78]. In addition there were two studies which utilised mg/kg [79, 80]. While these two studies are able to be compared more easily with the animal studies, this difference in measurement means that they are not comparable to the other studies as the masses of the participants are not provided.

**Table 5:** Studies of cannabinoids for anxiety in humans

Study	Participants	Methodology	Duration	Outcome measure	Cannabinoid	Route	Results
D’Souza et al. 2004 [77]	22 healthy individuals (male: n = 14, female: n = 8), mean age = 29, who had been exposed to cannabis but had never been diagnosed with a cannabis abuse disorder	In a double-blind, randomised, and counterbalanced study, over three days, the dose effects of THC were characterized in healthy individuals who had been exposed to cannabis but had never been diagnosed with a cannabis abuse disorder.	﻿Volunteers participated in 1-3 test days, separated by at least 1 week. VAS scores were recorded 60 min before ingestion and at 10, 80 and 200 min post ingestion.	Visual Analog Scale for anxiety (VAS)	THC (2.5, 5.0 mg)	Intravenous	THC transiently increased VAS scores of ‘anxious’ and decreased VAS scores of ‘calm and relaxed’ in a dose dependent manner
D’Souza et al. 2008 [48]	30 frequent users of cannabis, (male: n = 21, female: n = 9), mean age = 24.8,	In a double-blind, randomized, placebo-controlled study over three days, the dose related effects of THC were observed in 30 frequent users of cannabis and 22 healthy controls.	Volunteers participated in 1-3 test days, separated by at least 1 week. VAS scores were recorded 60 min before ingestion and at 10, 80 and 200 min post ingestion.	Visual Analog Scale for anxiety (VAS)	THC (2.5, 5.0 mg)	Intravenous	THC transiently increased VAS anxiety scores in both groups. However, frequent users showed smaller increases in anxiety than controls.
Bergamaschi et al. 2011 [82]	6 males and 6 females were placed in each group. The mean age for each group was between 22.9 and 24.6.	Participants with SAD and an additional 12 controls were blindly allocated to receive CBD or placebo 1.5 h before a simulation public speaking test. At six time points during the test Visual Analogue Mood Scale, and Negative Self-Statement scale, and physiological measures were taken.	Baseline measurements were taken, followed by ingestion of CBD or placebo. Pretest measurements were made 80 min after ingestion and post-test measurements were made 15 and 35 min after the end of the speech.	Visual Analog Scale for anxiety (VAS)	CBD (600 mg)	Oral	CBD administration resulted in significantly reduced anxiety, cognitive impairment and discomfort, and significantly decreased hyper-alertness in anticipatory speech. The CBD group and the healthy controls had similar response profiles.
Crippa et al. 2011 [83]	10 males with of a mean age of 24.2	Participants with SAD who were given CBD or placebo, in a double-blinded crossover manner. Regional cerebral blood flow activity in the brain was then compared	Subjective ratings were made at five different time points: 30 min before drug ingestion, at the time of ingestion and at 60, 75 and 140 min (or after SPECT procedure) after ingestion	Functional neuroimaging, Statistical parametric mapping	CBD (400 mg)	Oral	Compared to the placebo CBD produced significantly lower subjective anxiety and modulated blood flow in the left parahippocampal gyrus, hippocampus, and inferior temporal gyrus, and right posterior cingulate gyrus
Zuardi et al. 1982 [18]	8 physically and mentally stable volunteers (male: n = 6, female: n = 2), mean age of 27.	Participants received in a double-blind procedure, THC, CBD or both cannabinoids together across five experimental sessions, separated by a minimum interval of one week.	Each volunteer participated in five experimental sessions, separated by a minimum interval of 1 week.	State-Trait Anxiety Inventory (STAI)	THC (0.5 mg/kg) CBD (1.0 mg/kg) THC + CBD (0.5 mg/kg + 1.0 mg/kg)	Oral	THC produced a large increase in anxiety. This was particularly antagonized when CBD was administered simultaneously
Fusar-Poli et al. 2009 [86]	15 healthy males, who had used cannabis 15 times or less in their life with a mean age of 26.67	THC, CBD or a placebo were administered in a double-blind, randomised manner. Functional magnetic resonance imaging (fMRI) was used to observed the brain while participants viewed images of faces that implicitly intended to induce varying levels of anxiety.	Subjects were studied on 3 separate occasions, each separated by a 1-month interval. Either THC, CBD or a placebo was ingested before each session.	State-Trait Anxiety Inventory (STAI), VAMS anxiety and tranquilization subscale	THC (10.0 mg) CBD (600 mg)	Oral	THC resulted in increased anxiety being noted in the STAI score. Though CBD did not significantly change this rating, there was a trend (P = 0.06) for reduction in anxiety following CBD administration on the VAMS anxiety and tranquilization subscale.
Peters et al. 1976 [79]	Two groups, each consisting of 5 males and 5 females aged between 21 and 34. One group reported their marijuana use did not exceed twice per month over the past 3 to 4 years. The other group, reported using marijuana twice or more weekly during the same period.	THC was administered in a randomised, double blind procedure. The participants then filled out a 270 item questionnaire (SDEQ)	All 20 subjects were administered THC or a placebo on four separate occasions separated by at least 1 week. The SDEQ was undertaken 4.5 h after ingestion.	Subjective Drug Effects Questionnaire (SDEQ)	THC (0.2,0.4, 0.6 mg/kg)	Oral	THC was found to have a profound anxiogenic effect, with participants stating that they felt more tense, jittery and less in control
Karniol et al. 1974 [78]	40 male volunteers aged between 21 and 34. 22 stated that they had previous occasional use of cannabis	In a double-blind procedure, healthy male volunteers were assigned to one of eight experimental groups and in a double-blind procedure received THC, CBD or a mixture of both cannabinoids.	Pulse rate and a time production task (asked to produce a 60-sec interval, 10 times), was repeated before and after ingestion. At at 55, 95, 155 and 185 min after ingestion, subjects were asked questions about their feelings and sensations.	Reported anxiety and panic	THC (30.0 mg) CBD (15.0, 30.0, 60.0 mg) THC + CBD (30.0 mg + 15.0 mg, 30.0 mg + 30.0 mg, 30.0 mg + 60 mg)	Oral	THC provoked strong reactions, with four of the five subjects scoring their anxiety level as the maximum grade of four. CBD produced anxiogenic effects in two of the fifteen subjects. When CBD was given together with THC, the anxiogenic effects of THC were reduced.

Evidence of THC’s potential anxiolytic effects in humans, was first published in 2004. The study sample size consisted of 22 healthy individuals who had previously used cannabis, but had never been diagnosed with a cannabis abuse disorder [77]. In a 3-day, double-blind, randomised procedure, 22 volunteers received 2.5 or 5 mg of THC. They were asked to score their feelings using the Visual Analog Scale for anxiety (VAS-A) [81]. The results showed a statistically significant increase in VAS-A scores of ‘anxious’. This was observed to occur in a dosage-dependent manner, yet there were no statistically significant changes in the VAS-A scores for panic.

In a follow up US study, the same methodology was applied to people who were frequent users of cannabis [48]. The researchers aimed to determine if this frequent use offers protection from or tolerance to the effects of THC. Thirty frequent users were compared to 22 healthy volunteers, who acted as the control. Once again, a correlation between the dosage given and the VAS scores for anxiety was observed with VAS anxiety scores transiently increasing in both groups. It was noted that those who frequently smoked cannabis displayed significantly smaller increases in anxiety than controls.

Converse to the anxiogenic effects of THC, CBD appears to have the opposite effect. In Bergamaschi et al. (2011) [82], participants with social anxiety disorder (SAD) and an additional 12 controls were blindly allocated to receive CBD or placebo 1.5 h before a simulation public speaking test. The Visual Analogue Mood Scale (VAMS), Negative Self-Statement scale, and physiological measures were taken at six time points during the test. CBD administration resulted in significantly reduced anxiety, cognitive impairment and discomfort, and significantly decreased hyper-alertness in anticipatory speech. Further, Crippa et al. (2011) [83], observed regional cerebral blood flow activity in the brain of participants with SAD who were given CBD or placebo. CBD was found to modulate blood flow in the left parahippocampal gyrus, hippocampus, and inferior temporal gyrus, and right posterior cingulate gyrus. In addition, participants who received CBD reported significantly lower subjective anxiety than those who received a placebo.

Another two studies utilised Spielberger’s State-Trait Anxiety Inventory (STAI) to measure anxiety [18, 84]. In the first, participants participated in five experimental sessions where they received 0.5 mg/kg THC with the STAI being conducted at the start of the first and last experimental session [80]. In the second study this was done at baseline and 1,2, and 3 h post administration with 10 mg THC [84]. In both cases, an increased STAI score was noted. Further, it was found that both the STAI and the VAMS scores were significantly increased following THC intake relative to intake of a placebo. When CBD was administered alongside THC, this anxiogenic effect appeared to be reduced [18]. When CBD was given by itself, there was no change in the STAI score compared to the baseline. However, a possible reduction in anxiety was evidenced in the results of the VAMS anxiety and tranquilization subscale [84] Compared with placebo, CBD administration did not significantly change any of the subject ratings.

Another similar study, used a differing assessment, the Subjective Drug Effects Questionnaire (SDEQ) [79]. Ten frequent and 10 occasional cannabis users received doses of 0.2, 0.4, and 0.6 mg/kg THC. THC was found to have a profound anxiogenic effect, with participants stating that they felt increasingly more tense, jittery and less in control as the dose was increased. Karniol et al. (1974) [78] also reported a strong anxiogenic reaction as a result of THC administration with subjects expressing that the feeling of anxiety sometimes reached a near panic state. Further, four of the five subjects gave this feeling as the maximum grade possible in this study. In this case, 30 mg of THC was administered. This study also administered various doses of CBD (15.0, 30.0, 60.0 mg) to participants. Anxiety was reported by only two of the 15 subjects. When CBD was administered with THC, the anxiogenic effects of the latter were reduced.

## Discussion

### Data synthesis

The overall pattern of human clinical data supports consistent anxiogenic effects from THC, while CBD shows a consistent anxiolytic effect. In combination with CBD, the anxiogenic effect of THC has been shown to be decreased. However, further investigation is needed to categorically affirm this effect. Based on this data, it would imply that cannabis preparations higher in CBD and lower in THC cannabis would be most successful in treating anxiety. However, some survey data does not support this, with a preference for high THC cannabis being of greater interest to consumers for addressing affective symptoms. Further to this, only a very small percentage in surveys reported severe or intolerable side effects of using cannabis for their symptoms [51]; and in general, whole cannabis tends to have a much higher THC:CBD ratio. The epidemiological data is in contrast to the findings of the clinical trials.

These discrepancies could be due to the fact that while a substantial number of patients cross-sectionally report using cannabis and related products to treat anxiety symptoms or disorders, it has not been firmly established whether this anxiety occurred before or as a result of the cannabis usage [16, 85]. As epidemiological research largely relies on anonymous surveys, the composition of the cannabis being used is unable to be confirmed. It is known however, that between 1995 and 2015 there has been a 212% increase in THC content in the marijuana flower [86]. It is also known that plants producing high levels of THC are incapable of producing much CBD [86]. Thus, recent studies looking at whole cannabis consumption in theory should provide a relatively reliable source of information regarding the anxiogenic and/or anxiolytic properties of THC. Our review also highlights the lack of data from jurisdictions where cannabis is not legal, as most of the included studies are based on surveys by those living in certain states in the US or Canada where medicinal use is legal. An important consideration to note when assessing the epidemiological data is that many studies are based on self-reported effects from participants who are purposively using cannabis for their anxiety, and thus due to the sample bias, conclusions must be tempered.

In respect to the animal model research, there is strong evidence suggesting that an anxiolytic effect occurs after the administration of a small acute dose of CBD [60, 61, 63]. Results however differed depending on whether CBD was acutely or chronically administered, as well as the animal model used. This was demonstrated by Rubino et al. (2007) [67] and Schleicher et al. (2019) [70], who both observed no change in anxiety behaviour in the open field test, but significant changes in behavior in the elevated plus maze.

As the present data indicates, no clear conclusion can be drawn from the preclinical studies of acute administration of THC. This could in part be due to the types of animal model being utilised. For example, Onaivi et al. (1990) [19] found that in an elevated plus maze, THC induced both in rats and in mice, an increased aversion to the open arms of the elevated plus maze; but this effect was approximately three times greater in rats than in mice. Thus, while the two predominant tests for rodents are the elevated plus maze and the light–dark box, the results are difficult to compare as rats and mice may react differently to the test paradigm. This suggests that physiological parameters such as the cardiac conditioned response used by McLendon et al. (1976) [65] might be a more accurate measure as it relies much less on human observation.

In humans, research has also shown that the anxiogenic effects of THC are greater among infrequent or non-users relative to frequent users [16], and ﻿high potency THC in cannabis products in particular, are thought to induce the development of psychotic-like symptoms or overt psychosis in vulnerable individuals. ﻿Similarly, intoxication by low-dose CBD has been found to be particularly prominent in infrequent cannabis users [38]. Further, it has been observed in early 1970s research that individuals who were anxious before receiving it became less anxious under the influence of cannabis (note that potentially far lower THC preparations would have been used). Conversely, non-anxious persons became more anxious [87]. In an animal model, Long et al. (2010) [69] found that differences were observed amongst mice depending on the day in which they were tested, which suggests that the length of time over which the treatment is given also effects the anxiolytic and anxiogenic properties. Kasten et al. (2019) [72] also observed inconsistencies across the groups investigated, with adolescent male mice performing differently to adult male mice, which in turn performed differently to adolescent female and adult female mice. This clearly shows that age, sex and background of exposure may have an impact on how an animal or human reacts to THC or CBD inoculation, something which was found by Cuttler et al’s. (2016) [59] epidemiological survey, where different results were reported depending on the sex of the person responding.

Differences in methodologies and limitations of data provided across the studies reviewed, further reduces our ability to draw strong conclusions. This includes the irregularities in doses given, where some studies used mg/kg and others mg only, and different administration methods being used. Most acute studies using THC employ an oral or inhalation route of administration [77]. Oral administration delays the onset of effects by 30 min to two hours, produces lower peak plasma levels, and prolongs the action of the THC compared to the inhaled or intravenous route [88, 89].

In summary, the human clinical studies using acute THC consistently produced an anxiogenic effect, while studies using CBD and epidemiological studies of whole plant cannabis in anxiety disorders showed an anxiolytic effect. This is surprising as the doses of CBD that have been shown to have therapeutic effects are far lower than what is commonly found in cannabis plant matter, such as that which is being used by the majority of participants surveyed in the epidemiological studies [38]. Furthermore, these findings have not been reliably replicated in animal studies, and further larger human RCTs are required for stronger validation.

### Development of optimal anxiolytic cannabinoid therapies

Pharmacological treatment of anxiety relies on our understanding of the neurobiological interactions responsible [90]. While there are various different targets, the endocannabinoid system has, in recent years, increasingly been attributed with the control of stress, anxiety and fear. Endocannabinoids appear to modulate this system as well as the dopamine system, and hypothalamo-pituitary-adrenocortical axis [46, 91].

Though several classes of synthetic CB receptor agonists have been developed, these alternatives are high-potency CB_1_ receptor activators which elicit pronounced psychotropic effects, something which has seen them recently revoked across most Western countries. THC on the other hand, is a partial agonist at the CB1 receptor [38, 90], while CBD acts with low-affinity on the CB1 and CB2 receptors [38]. Cannabis, as a substrate of the CB_1_ and CB_2_ receptors in the endocannabinoid system, is therefore a prime substance for investigation.

However, research has been limited given the controversial legal history surrounding cannabis. Policies are rapidly evolving, and access to cannabis and cannabinoid products is increasing worldwide [38], with it now being decriminalised or permitted for medical purposes in many countries [92]. In Australia however, this change only took place in 2016. Prior to this it was considered a schedule 9 drug and so research into its medical use has been highly restricted [93]. As such this is still an emerging field.

With ongoing clinical research into the use of cannabis for anxiety, it is likely that optimised cannabinoid ratios of THC and CBD will eventually be better understood. Various software programs in use by the general public (e.g. Strainprint Technologies, Releaf etc.) may also be of value to researchers tackling this challenge. Apps such as these are able to track patient symptoms and collect data on the specific cannabis dosage form, cannabinoid ratios and particular cannabis products used for certain diseases, conditions or symptomatic relief.

These two constituents may only be part of the story, and continuing research into the pharmacological activity of the cannabinoids themselves may reveal that THC and CBD are not the only cannabinoids of clinical interest in anxiety. Notwithstanding the academic appetite for cannabinoid research, an often-overlooked phytochemical class, such as the terpenes/terpenoids, has also shown significant anxiolytic action. D-limonene and linalool, whilst not exclusively found in cannabis, have demonstrated anxiolytic activity; the former via the 5HT1A receptor [4, 94, 95]. As such, specific chemovars of cannabis with higher expression of these terpenes may be of greater clinical interest, particularly when paired with higher CBD concentrations. With such a complex chemistry extent in the *Cannabis* genus, it is plausible that many phytochemicals could be contributing to anxiolytic activity, likely interacting with numerous receptor types. ﻿ Further, as previously mentioned, some research has shown that the adverse effects of THC, may be dose dependent and are potentially decreased by low doses of CBD [38]. Hence, further research into these interactions would contribute greatly to this area.

Lastly, each individual using cannabis is also unique, making the study of pharmacogenomics an important aspect of ongoing cannabis research [96]. Variability in cannabinoid receptor genes, transporter genes and pharmacokinetic drug metabolism [97], such as that observed in the Cytochrome P450 system, are important factors for consideration. Further investigation of single nucleotide polymorphisms (SNPs), in particular of fatty acid amide hydrolase (FAAH), may potentially affect individual responses to CBD, and is another worthy research pathway in the future [96].

## Conclusion

The results of this review suggest that there is tentative support based on epidemiological surveys and clinical studies showing that whole cannabis and CBD may have a beneficial role in anxiety disorders (for certain candidates in this population). In contrast, for isolated THC, acute human studies consistently show an anxiogenic effect. However, animal studies show that there may be potential for THC to be used as an anxiolytic, if given at the right dose for the patient, and that this may require gradual titration to ameliorate initial anxiogenic effects. Further to this, such an approach may be assisted via the co-administration of CBD, other cannabinoids or terpenes found in the cannabis plant which have yet to be studied substantially.

Further human studies are needed to establish consistency in the results, therapeutic thresholds, and dosage required for cannabinoid therapies to produce an anxiolytic effect in humans, and further research on cannabinoids and terpenes may yield a more optimised anxiolytic formulation.

## Data Availability

Not applicable.

## Abbreviations

CBD: Cannabinol
fMRI: Functional Magnetic Resonance Imaging
LD: Light–dark (box)
OF: Open Field (test)
SDEQ: Subjective drug effects questionnaire (SDEQ)
STAI: State-Trait Anxiety Inventory
THC: Tetrohydrocannabiniol/Δ^9^-tetrahydrocannabinol
VAMS: Visual analogue mood scale
VAS: Visual analog scale
